# Impact of analytic provenance in genome analysis

**DOI:** 10.1186/1471-2164-15-S8-S1

**Published:** 2014-11-13

**Authors:** Shatavia S Morrison, Roman Pyzh, Myung S Jeon, Carmen Amaro, Francisco J Roig, Craig Baker-Austin, James D Oliver, Cynthia J Gibas

**Affiliations:** 1 Department of Bioinformatics and Genomics, University of North Carolina at Charlotte, Charlotte, North Carolina 28223, USA; 2 Department of Microbiology and Ecology, University of Valencia, Valencia, Spain; 3 Centre for Environment, Fisheries, and Aquaculture Science, Weymouth, Dorset, UK; 4 Department of Biology, University of North Carolina at Charlotte, Charlotte, North Carolina 28223, USA

## Abstract

**Background:**

Many computational methods are available for assembly and annotation of newly sequenced microbial genomes. However, when new genomes are reported in the literature, there is frequently very little critical analysis of choices made during the sequence assembly and gene annotation stages. These choices have a direct impact on the biologically relevant products of a genomic analysis - for instance identification of common and differentiating regions among genomes in a comparison, or identification of enriched gene functional categories in a specific strain. Here, we examine the outcomes of different assembly and analysis steps in typical workflows in a comparison among strains of *Vibrio vulnificus*.

**Results:**

Using six recently sequenced strains of *V. vulnificus*, we demonstrate the "alternate realities" of comparative genomics, and how they depend on the choice of a robust assembly method and accurate *ab initio *annotation. We apply several popular assemblers for paired-end Illumina data, and three well-regarded *ab initio *genefinders. We demonstrate significant differences in detected gene overlap among comparative genomics workflows that depend on these two steps. The divergence between workflows, even those using widely adopted methods, is obvious both at the single genome level and when a comparison is performed. In a typical example where multiple workflows are applied to the strain *V. vulnificus *CECT 4606, a workflow that uses the Velvet assembler and Glimmer gene finder identifies 3275 gene features, while a workflow that uses the Velvet assembler and the RAST annotation system identifies 5011 gene features. Only 3171 genes are identical between both workflows. When we examine 9 assembly/ annotation workflow scenarios as input to a three-way genome comparison, differentiating genes and even differentially represented functional categories change significantly from scenario to scenario.

**Conclusions:**

Inconsistencies in genomic analysis can arise depending on the choices that are made during the assembly and annotation stages. These inconsistencies can have a significant impact on the interpretation of an individual genome's content. The impact is multiplied when comparison of content and function among multiple genomes is the goal. Tracking the analysis history of the data - its analytic provenance - is critical for reproducible analysis of genome data.

## Background

Next generation sequencing has revolutionized the study of microbial genomics. To handle the millions of sequence read fragments produced by the next gen platforms, a variety of assembly approaches have been developed[1-3]. In most instances the assembler produces a set of contigs or scaffolds, which still leaves the genome in pieces. It is no longer common to completely finish and close a newly-sequenced genome. Usually, we evaluate the "success" of the assembly with two metrics: the number of contigs produced and the N50 value. Lower contig counts and higher N50 values are considered optimal. However, Parra *et al*. [4] and others [5] reported that choosing assemblies with higher N50 values frequently results in conserved genes going undetected in benchmark studies. If a gene is missed due to errors at the assembly stage it will not be annotated, leading to inconsistencies in downstream analyses.

There have been several efforts to assess the quality of assemblies produced by *de novo *methods. GAGE [6] and the Assemblathon [7] projects provided gold-standard data sets and an environment for peer evaluation of assembly methods. Recently, next generation sequence assemblers were evaluated on bacterial datasets in the GAGE-B study. Magoc *et al*.[8] showed that a single library prep and deep (100x-250x) sequencing coverage is sufficient to capture the genomic content of most bacterial species, but demonstrated wide variation in the assemblies produced by different methods.

Analysis of genomes does not stop at assembly, however. There exist a wide range of methods for annotation of the assembled data. Genome annotation includes identification of the gene sequences within a contig, and assignment of function based on similarity to known genes or sequence patterns. *Ab initio *gene finders and methods for functional assignment each have their own associated uncertainty, and results from one method are unlikely to agree completely with those from another[5]. Assembly and annotation are the two major components of the bacterial genomics workflow, and there are an astonishing number of combinations of methods that can be used to carry out just these two steps.

**Figure 1:**
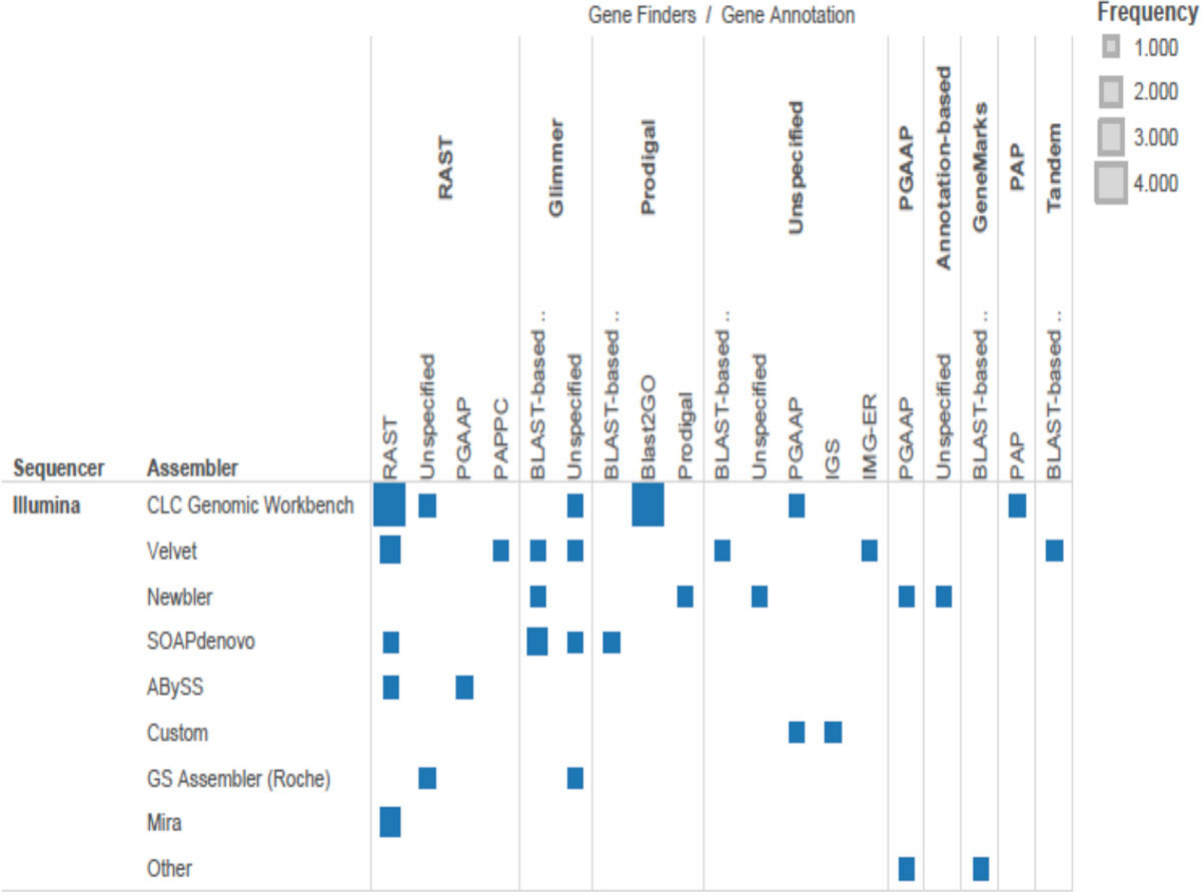
**Crosstab map of frequency levels of assembler and annotation method applied to Illumina data**. Figure shows the frequency of the number of times a particular combination of assembler and annotation method was used in 40 Genome Announcements from the September 2012 Vol. 194, Issues 17 and 18 of Journal of Bacteriology and January and February 2013 Vol. 1., Issue 1 of Genome Annoucements.

When we survey the literature in microbial genomics, we find that investigators depositing microbial sequences have not come to a consensus on the best pipeline for genome analysis. Several different assemblers are in common use. Annotation methods may include anything from simply comparing the genome to a reference by using BLAST, to using *ab initio *genefinders, to using integrated annotation pipelines provided by sequencing centers. Despite over a decade of literature on the performance of *ab initio *genefinders and annotation pipelines[9-12] nearly any reasonable workflow seems able to pass peer review (Figure 1), and so the genome annotations found in the public databases vary widely in analytic provenance. Especially in the absence of reference genomes and bench work validation, the proliferation of analysis options can lead to inconsistencies (comparing apples to oranges) and ultimately to errors in biological interpretation. It is not possible to distinguish a true target, such as a gene that differentiates one genome from its near relatives, from an artifact introduced at the assembly or annotation steps. Yet investigators often seem to remain unaware of the impact of their choices, and how the selection of Glimmer[13] rather than GeneMark [14] (for example) may result in a greatly altered story when they begin to analyze the apparent content of a newly sequenced genome. Figure 1 is a summary of the major elements of current genomic workflows based on a census of 2013 bacterial genome announcements in recent issues of the journal GenomeA (American Society of Microbiology) [15].

In recent years, the biomedical research community has increasingly recognized the failure of many studies to achieve reproducibility [16] in data analysis protocols. In experiments using NGS data, which rely entirely upon computational analyses for interpretability, the ability to trace the history of and reproduce data analysis is especially critical [17,18]. Innovation in this domain is rapid and is ongoing, and best practices for reproducibility in bioinformatics are increasingly widely discussed[19]. The concept of workflow, originally used to describe business processes, began to be used more broadly in bioinformatics with the advent of high throughput sequencing in the early 2000s [20]. Systems such as Galaxy [21], Taverna [22], and MOLGENIS[23], among others, have made reproducible workflows more accessible to users of bioinformatics software, and many workflow systems now include the means for tracking analytic provenance,[19]but it is clear from our survey of the literature that these innovations have yet to completely penetrate to end users of assembly and annotation methods for microbial genome sequencing.

In this study, we assess the scope of the data interpretation problem caused by variation in pipeline choices. Starting with five *V. vulnificus *strains for which paired-end Illumina sequence was collected, and one *V. vulnificus *genome with a high quality finished sequence that has been continually revised and updated [24], we apply well-regarded assembly and annotation methods, in different combinations, to the data. We have chosen to focus on only a few of those most popular methods in each category, because workflow construction from multiple options is a combinatorial problem. The case study data demonstrate the influence of choices made during the assembly and annotation stages on biological interpretation of newly sequenced genomes. *Vibrio vulnificus *is a bacterium commonly found in estuarine waters and mollusks. It is responsible for 95% of all deaths resulting from seafood consumption in the United States [25]. There are both clinical isolates and environmental genotypes associated with this bacterium, making it a prime candidate for comparative genomics study. In the present study, we demonstrate the direct impact of parameter and method choices on the biologically relevant products of a comparative genomics analysis among strains of *Vibrio vulnificus*. Comparative analysis of gene content and function is a highly relevant case study, as this analysis is a popular protocol among microbiologists, and has been shown to be more effective than MLST for bacterial strain characterization [26]. The results highlight the influence of the assembly and annotation pipeline on comparative content and function analysis, and emphasize the need for contributors of genomic data to provide complete information about the analytic provenance of their assembled and annotated genomes, and for consistent workflows, justified by benchmark testing where possible, to be used throughout a project. Workflows used in this analysis were constructed in the Taverna workflow system, and are available as a workflow pack at http://MyExperiment.org. [http://www.myexperiment.org/packs/625.html].

## Results

### Workflow dependent outcomes in a simulated assembly case

As a basis for choosing an appropriate analysis pipeline for newly sequenced *V. vulnificus *genomes, we first generated simulated read data from the genome of *V. vulnificus *CMCP6. This genome was initially sequenced using Sanger sequencing and a traditional genome finishing approach in 2003, [27] and was partially sequenced and completely reannotated in 2011[24]. While the original annotation relied primarily on a combination of *ab initio *genefinders, the subsequent reannotation used additional information from closely homologous genomes and public databases of curated gene sequence patterns. The published sequence and annotations for *V. vulnificus* CMCP6 are still not exhaustively validated by transcriptome data, but they are the most heavily curated of the available *Vibrio vulnificus *genome annotations, and therefore we use them as the frame of reference for evaluating different approaches to assembly and annotation.

We performed *de novo *sequence assemblies of the simulated data with Velvet (V), ABySS (A), and SoapDenovo (S). GeneMark.hmm (GeneMark)[14] and RAST[28] were then used to identify gene sequences for each contig set. We used OrthoMCL[29] with a stringent similarity cutoff to cluster predicted genes with their counterparts in the 2011 *V. vulnificus *CMCP6 annotation.

The contig counts observed were 205, 144, and 269 for the V, A, and S assemblies, respectively. Table 1 summarizes gene counts obtained for each assembly followed by each gene annotation method, for the simulated *V. vulnificus *CMCP6 genomes. To avoid ambiguity, the percentage of genes recovered refers only to predicted genes, which clustered uniquely with one gene in the reference annotation. Less than 1% of predicted genes cluster with apparent paralogs in the reference genome when clustered at a 95% threshold. The results presented in Table 1 suggest that, while the Velvet assembler[1] does not assemble the simulated data into the smallest number of contigs, it produces the most accurate assembly of the simulated *V. vulnificus *CMCP6 data. Velvet, in combination with the GeneMark[14]*ab initio *genefinder, may produce the best results on novel *V. vulnificus *sequence data. This type of simple two-step workflow is representative of genome analysis workflows found in the genome announcements surveyed in Figure 1. However, it should be noted that the best-performing workflow still resulted in a loss of over 200 previously annotated genes, when reanalyzing simulated *V. vulnificus *CMCP6 data.

**Table 1:** Assembly and Annotation of *V. vulnificus *CMCP6.

Assembly method	Velvet	ABySS	Soap
# of contigs	205	144	269

Assembly+RAST performance			

# of genes predicted	4684	5095	4720

# of genes with match in CMCP6	3890	3777	3863

% of known genes recovered	91.8%	89.2%	91.2%

Assembly+Genemark performance			

# of genes predicted	4761	5051	4833

# of genes with match in CMCP6	4019	3754	3844

% of known genes recovered	94.9%	88.6%	90.7%

### Workflow dependent outcomes on novel genome data

The published *Vibrio vulnificus *genomes are mainly composed of 2 circular chromosomes, and some are known to have plasmids. The size of the *V. vulnificus *genome is estimated at 5.6 Mb-5.8 Mb of DNA, and this size is consistent among known strains. The newly sequenced isolates *V. vulnificus *CIP8190, CECT5198, CECT4606, CECT5763, and CECT4886 are all known to have 2 chromosomes and 2,3,1,2, and 2 plasmids, respectively. Table 2 describes each genome used in this study and its genomic characteristics, as well as the number of sequence reads available for each genome.

**Table 2:** Genomic Characteristics of *Vibrio vulnificus *CMCP6, CIP8190, CECT5198, CECT4606, CECT5763, and CECT4866.

Genomic Characteristic	CMCP6	CIP8190	CECT5198	CECT4606	CECT5763	CECT4866
Biotype	1	2	2	2	2	2

Genotype	C	C	E	E	E	C

Chr Number	2	2	2	2	2	2

Plasmid Number	None	2	3	2	2	2

Average G+C content	46.6 %	46.5%	46.5%	46.2%	46.3%	46.5%

# of reads generated	6620286^*^	26869740	14366914	23523786	18852452	33792718

N50 for Velvet	196375	71778	60906	316446	51991	65142

N50 for ABySS	187671	57867	66098	154882	54273	64876

N50 for Soap	196396	71391	62139	165040	52087	65144

**Figure 2:**
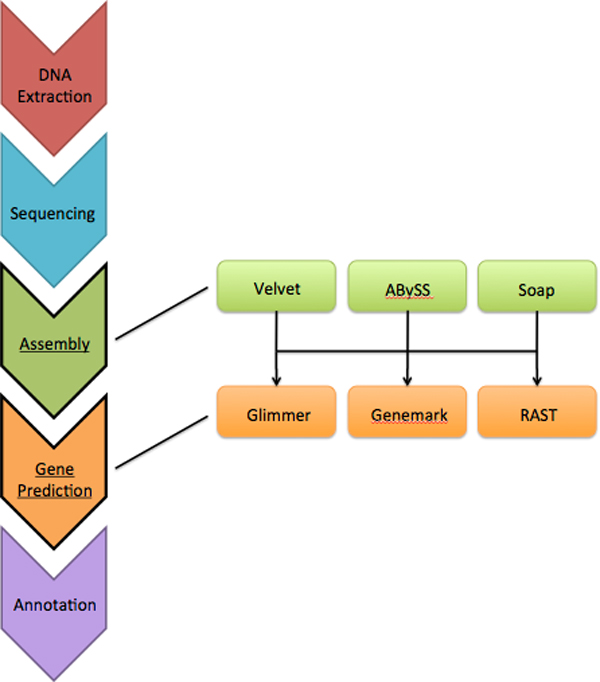
**Workflow framework of assembler and annotation methods**. Figure shows the assembly and annotation software application applied to each of the *V. vulnificus *strains included in this study.

Our analysis here is primarily focused on the performance of the assembly and annotation steps typically used during the construction of a draft genome. Biological findings for these genomes will be the focus of another manuscript, currently in preparation. Using the workflow framework shown in Figure 2, we assembled contig sets and annotation sets for each *V. vulnificus *strain. After the removal of sequence reads containing 'N' characters, and random sampling of read pairs to obtain 100x genome coverage based on the Lander Waterman statistic[30], there were 11,400,000 paired end reads in the final read sets for each of the newly sequenced strains. The same coverage depth was simulated for *V. vulnificus *CMCP6.

**Figure 3:**
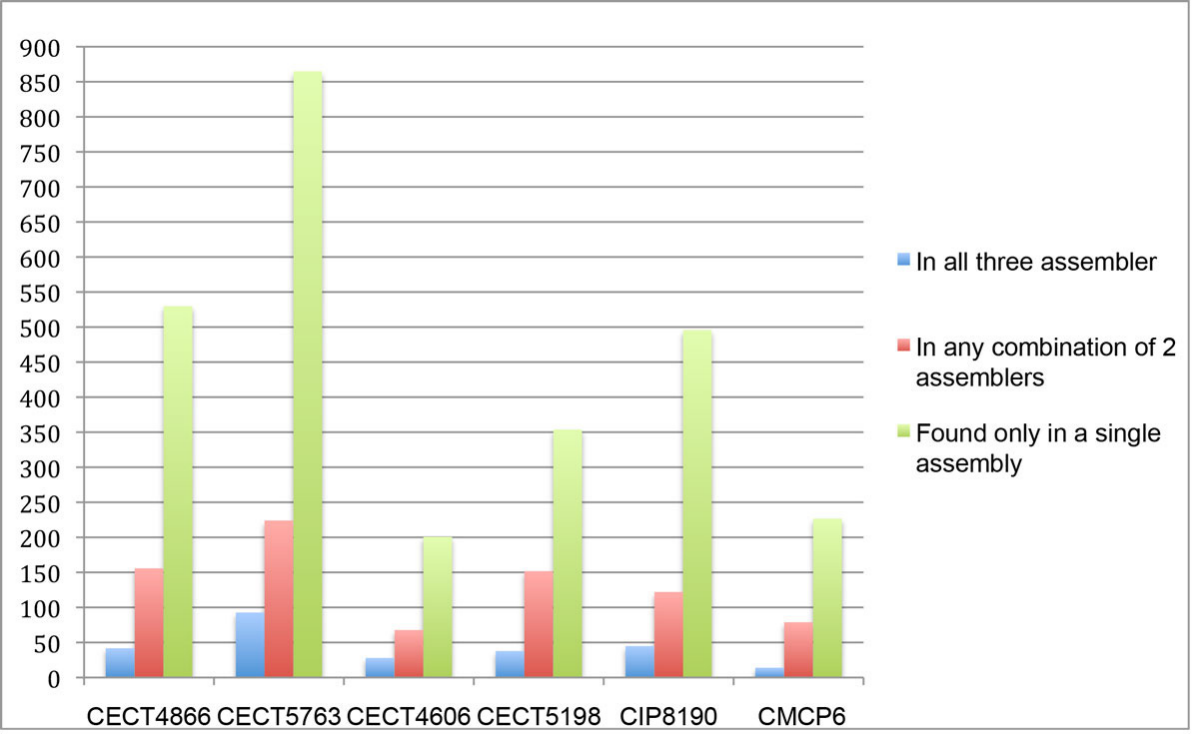
**Comparison count of highly conserved contigs for *V. vulnificus *CMCP6, CECT4606, and CECT5198**. Figure shows the counts of the number of contigs that were identified by 1 assembler, by any combination of 2 assemblers, and by all three assemblers.

Using the same *de novo *assemblers we applied to the simulated data set, we constructed contig sets ranging in size from 180-630 contigs for each of the input genomes. Table 3 summarizes the output of Velvet, Soap, and ABySS assemblies for each *V. vulnificus *strain. We then used MuMMer 2.3[31]to align the contig sets for each strain, using an all-against-all alignment to identify contigs that were similarly constructed between the assemblers. Contig pairs that exceeded coverage and sequence identity cut-offs of 95% were identified as similarly constructed. Figure 3 summarizes the conservation of contigs across assemblies. Although counts varied from genome to genome, we observed on average 43 contigs constructed by all three assemblers, 133 found by any combination of two of the three assemblers, and 445 contigs that were uniquely constructed by a specific assembler.

**Table 3:** Total number of contigs assembled for *V. vulnificus *CMCP6, CIP8190, CECT5198, CECT4606, CECT5763, and CECT4866.

Strain	Velvet	Abyss	Soap
*V. vulnificus *CMCP6	205	144	269

*V. vulnificus *CIP8190	284	364	507

*V. vulnificus *CECT5198	302	289	448

*V. vulnificus *CECT4606	129	148	267

*V. vulnificus *CECT5763	492	743	845

*V. vulnificus *CECT4866	404	366	519

**Figure 4:**
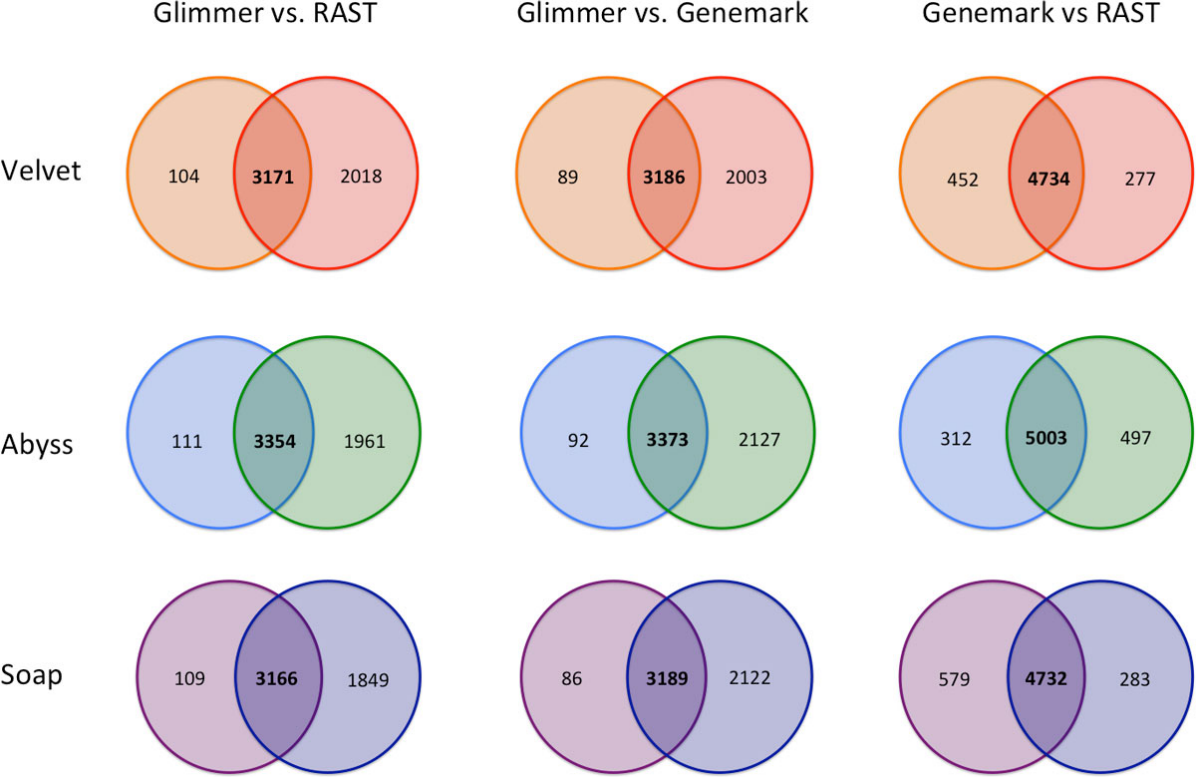
***Vibrio vulnificus *CECT4606 gene overlaps counts**. Figure shows we detail the number of gene overlaps between all possible assembly-to-annotation permutations for *V. vulnificus *CECT4606. Gene overlaps are defined as two annotated genes from different pipelines that have the same stop signals and strand orientation on the same contig sequence.

In our analysis of the novel *Vibrio vulnificus *genomes, we included the Glimmer3.0[13]*ab initio *gene-finding method in addition to GeneMark and RAST. Glimmer3.0 is demonstrated to be approximately 96% accurate in gene identification,[13]which is similar to the accuracy that we observed for GeneMark in the CMCP6 case study above. In Table 4, we summarize the gene predictions by each of the three prediction methods for each of the three assemblies constructed for each *V. vulnificus *strain. We find that RAST and GeneMark tend to identify more regions as putative genes sequences than Glimmer for these strains. However, this is not a case of simple over-prediction, since the Glimmer gene sequences are not strictly a subset of the predictions by other methods. As an example, in Figure 4 we detail the number of gene overlaps between all possible assembly-to-annotation permutations for *V. vulnificus *CECT4606.

**Table 4:** Total number of genes predicted for *V. vulnificus *strains included in this study.

A.)			
**Glimmer**	**Abyss**	**Soap**	**Velvet**

*V. vulnificus *CMCP6	3226	3042	3047

*V. vulnificus *CIP8190	3233	3030	3032

*V. vulnificus *CECT5198	3289	2973	2977

*V. vulnificus *CECT4606	3465	3275	3275

*V. vulnificus *CECT5763	3253	3079	3083

*V. vulnificus *CECT4866	3301	3024	3031

**B.)**			

**Rast**	**Abyss**	**Soap**	**Velvet**

*V. vulnificus *CMCP6	5095	4720	4684

*V. vulnificus *CIP8190	4963	4600	4623

*V. vulnificus *CECT5198	5021	4554	4563

*V. vulnificus *CECT4606	5315	5015	5011

*V. vulnificus *CECT5763	5038	4732	4752

*V. vulnificus *CECT4866	5035	4605	4631

**C.)**			

**Genemark**	**Abyss**	**Soap**	**Velvet**

*V. vulnificus *CMCP6	5051	4833	4761

*V. vulnificus *CIP8190	5084	4912	4787

*V. vulnificus *CECT5198	5187	4795	4710

*V. vulnificus *CECT4606	5500	5311	5189

*V. vulnificus *CECT5763	5489	5346	5062

*V. vulnificus *CECT4866	5243	4931	4839

**Figure 5:**
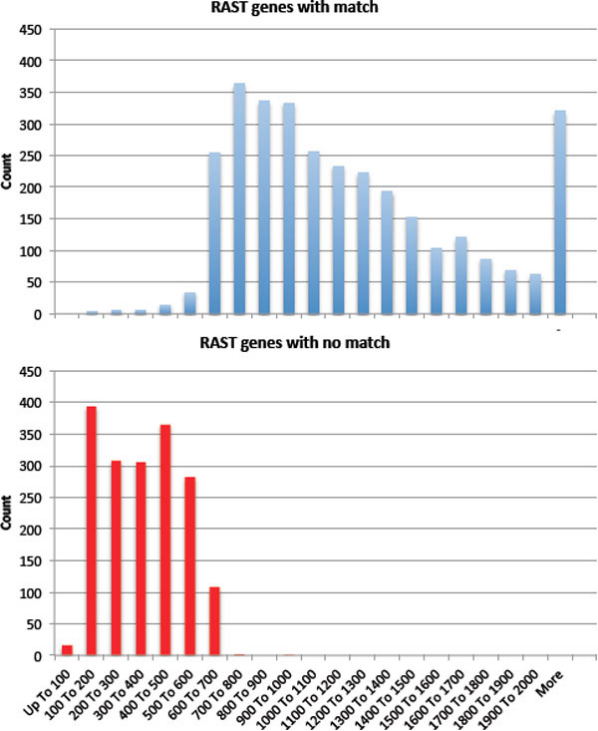
**Distribution of gene lengths for gene predicted using RAST in *Vibrio vulnificus *CECT4606 with the SoapDenovo assembly**. Consensus gene lengths, which have a match in the alternate annotation generated using Glimmer are plotted in blue, while non-consensus gene lengths are shown in red.

Figure 4 summarizes the gene overlaps for *Vibrio vulnificus *CECT4606 datasets for different genefinders applied to assemblies. Gene overlaps are defined as two genes identified by different pipelines, which have the same stop signals and strand orientation on the same contig sequence. In prokaryotes, *ab initio *genefinder predictions are known to be least reliable for very short genes[32]. As an example, in Figure 5, we show the distribution of gene lengths for consensus and non-consensus genes in a case were the RAST and Glimmer genefinders were both applied to the genome of *V. vulnificus *CECT4606, with the SoapDenovo assembler. Genes of length 500 and below are nearly entirely non-consensus genes, while genes above 700 in length are nearly entirely in consensus between the two methods. It is in the region between 500 and 700 nucleotides where potentially ambiguous cases are found, involving several hundred genes. Glimmer tends to predict fewer genes that are outside the common "core" of predictions produced by all three genefinders. It is possible that this reflects greater accuracy, or it may be that Glimmer alone is more conservative in its gene-identification model. RAST (which uses Glimmer in an initial annotation pass) and GeneMark both make, and agree upon, predictions that are excluded from the Glimmer prediction set. It is possible that these two methods are potentially capturing more species-specific genes.

### Workflow dependent outcomes in functional analysis

An archetypal result presented in genomic analyses is the categorization of genes into functional categories. This type of analysis is frequently used to draw conclusions about the energy sources an organism can use for survival, or about the genome's capacity to code for systems related to pathogenicity. To illustrate the impact of workflow choice on interpretation of functional content, we performed a comparative analysis among the results of six assembly-to-annotation workflows applied to the genome of *V. vulnificus *CECT4866, refer to Table 5. We used the GenoSets[33] analysis system to perform the comparison of analysis outcomes, treating the annotation set produced by each workflow as if it were an independent "genome".

**Table 5:** Workflow descriptions used in differential functional analysis of *Vibrio vulnificus *CECT4866.

Workflow Assignment	Assembly Type	Annotation Method	Number of genes
A	Velvet	Glimmer	3031

B	Velvet	Genemark	4839

C	Abyss	Rast	5035

D	Abyss	Glimmer	3301

E	Soap	Genemark	4931

F	Soap	Rast	4605

Each workflow's gene set was assigned Gene Ontology (GO) terms [34,35]as described in Cain *et al*., 2012[33]. GO categories and individual genes having functionality significant enrichment or depletion between the various annotation versions were identified using the Gene Ontologizer[36]. See additional file 1 which summarizes the complete GO enrichment set for each of the workflow combinations examined. We first compared annotations produced by a workflow that used the Velvet assembler, followed by either Glimmer or GeneMark. 134 genes appeared in the Glimmer predictions, but not in the GeneMark predictions, resulting in the appearance of statistically significant enrichment or depletion in two GO functional categories. Deoxyribose phosphate metabolic process and deoxyribose phosphate catabolic process p-values were 0.0066 and 0.0072, respectively. 120 genes were identified solely with GeneMark annotations. Use of GeneMark resulted in the appearance of enrichment in GO terms associated with response to stress and iron ion binding, with p-values at 5.99^E-12 ^and 0.0017, respectively. The GO terms associated with iron utilization are especially of interest in the context of *Vibrio vulnificus *genomics, because as a pathogen it is especially dangerous to hosts in a condition of iron overload[37]. Iron-protein binding and stress response are potentially regarded as factors contributing to *V. vulnificus*'s pathogenicity. Several studies have reported on the correlation between *V. vulnificus *infections and increased levels of iron in animal models and infected individuals[25,37,38]. Wright *et al*.[37] showed the injecting mice with iron prior to *V. vulnificus *infection significantly lowered the LD_50_. Amaro *et al*.[38] showed that after the injection of *V. vulnificus *to an iron-overload mice, they always died within a 48 hour period of inoculation. In this case, changing the assembly-to-annotation analysis pipeline results in a significant change in detected gene content, in a category that is directly relevant to the biology of the pathogen.

We next examined pipelines using the ABySS assembler followed by RAST or Glimmer. 1880 genes were unique to the RAST annotation. Of these, 132 significant GO enrichment terms were identified. In this set we find both iron-binding protein and terms associated with response to stress, again suggesting that the choice of assembly-to-annotation pipeline has the potential to significantly alter biological interpretation. Only 148 gene clusters were unique to the Glimmer set, and only 5 functional categories showed apparent statistically significant enrichment. Comparison of RAST and GeneMark annotations on a SOAPdenovo assembly resulted in approximately 10 statistically significant differences in functional content in either direction, although none of these categories were identified as significant to the biology of *V. vulnificus *in a previous study[39].

While these results are not conclusive, they indicate that at least in the case of *V. vulnificus*, RAST or GeneMark predictions may best reflect the presence of genes in key functional categories, known to be significant in the biology of these organisms.

### Workflow dependent outcomes in genome content comparison

**Figure 6:**
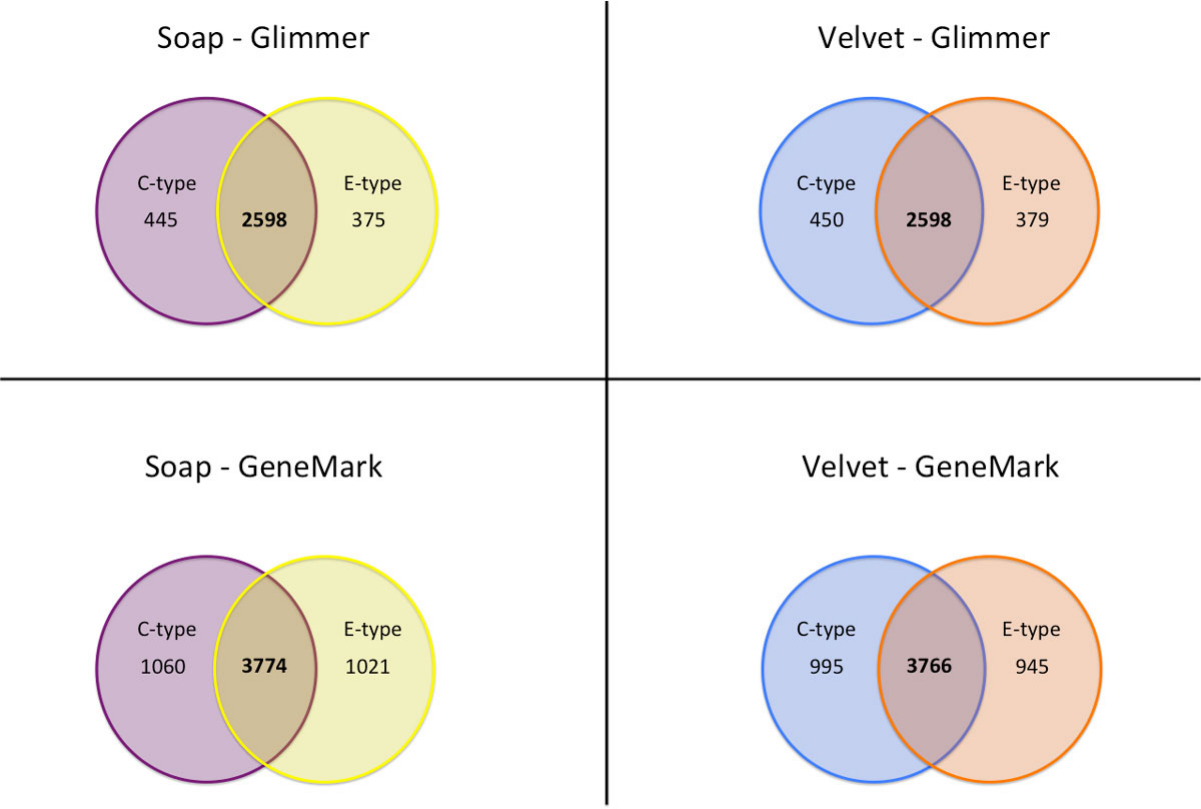
**Genome content comparison for *Vibrio vulnificus *CMCP6 and CECT5198**. Venn diagrams represent the differential and shared gene counts between *V. vulnificus *CMCP6 and *V. vulnificus *CECT5198 using the Velvet and SoapDenovo assemblies, each with Glimmer and GeneMark annotations.

Another archetypal figure found in nearly every comparative genomics analysis paper is the Venn diagram or its conceptual equivalent. The Venn diagram provides a convenient method to summarize what the microbiologist really wants to know: what is in strain (or species) A that makes it function differently from strain B? In Figure 6, we show the effect on this commonly-generated analysis product when different assembly-to-annotation pipelines are used to generate the input data. As an illustrative example, we performed gene content comparisons between *V. vulnificus *strain CMCP6 (clinical genotype) and strain CECT5198 (environmental genotype). In each comparison, the same assembly-to-annotation pipeline was used on each of the genomes being compared. We tested four combinations of assembler and genefinder. In Figure 6, we show that the majority of differences are seen when different annotation methods are used. In contrast, when different assemblers are used with the same annotation method, the number of differential genes are highly conserved. Given the large number of non-identical genes found when different pipelines are used on the same genome, as we saw in the previous examples, the result is as expected - the valuable biological "end product", the set of differentiating genes around which the biologist will build their scientific conclusions, can vary by dozens if not hundreds of members.

## Discussion

Many factors can have an impact on the assembly of next generation sequence data. Typical information captured about the provenance of sequence data focuses on laboratory procedures and conditions, as we see in the MIGS[40] standard for genomic data, or in the experiment information preserved in, for example, the NCBI's Gene Expression Omnibus[41]. However, assuming that samples were properly handled and prepared in the laboratory, and that procedures and conditions are consistent, there is still an entire layer of provenance information to be considered. Here, we have considered the analytic provenance of genome sequence data, that is, the computational steps that are executed to process the data and to attach features and functional information that allows for interpretation.

Despite an attitude on the part of researchers and publishers that microbial genome analysis is a solved problem, application of multiple assembly-to-annotation pipelines to the same data demonstrates that analysis outcomes are heavily dependent on pipeline choice. These choices carry forward into comparative content analysis and functional analysis of genomes, and have the potential to significantly impact scientific conclusions.

It is now typical to report on novel microbial genomes in terse genome announcements, abstract-style papers that give little information about parameterization and execution of bioinformatics processes. A survey of these typical papers shows that a wide variety of genome analysis pipelines using combinations of bioinformatics tools, from simple to sophisticated, will pass peer review. However, on closer examination typical pipelines do not produce identical or even similar results. And while in the hands of trained bioinformaticians, the pipelines we tested in this paper may be fine-tuned to produce somewhat more accurate results, the literature surveyed suggests that this is not what is happening "on the ground" in analysis of bacterial genomes. If the protocols outlined in recent genome reports are accurate, in many cases these protocols are no more complex than the simple one assembler, one genefinder workflows we have analyzed here.

## Conclusions

While in many cases, there is not a standardized set of assembly and gene annotation tools as well as pipeline workflows for novel genome assemblies and annotations available, we recommend that creators of microbial genome datasets consider the following strategies to ensure high quality, reproducible analysis. First, if possible, benchmark proposed analysis pipelines using simulated data derived from a high-quality genome sequence that is as closely related to the novel sequences as possible[42]. Second, maintain an awareness of the variability of assembly-to-annotation results. Perform parallel analyses and assess downstream results for pipeline dependence. Finally, maintain a detailed record of the analytic provenance of the secondary data generated from your raw sequence reads, including pipeline steps and parameters.

## Methods

### Genome sequencing

*V. vulnificus *strains were sequenced at The Genome Analysis Centre (TGAC) using the Illumina HiSeq2000 platform. Sequencing was carried out on pooled libraries, using pools of 12 strains in one lane of the Illumina HiSeq 2000, and producing on average 100 base pair paired-end reads.

### Sequencing simulation

*V. vulnificus *CMCP6 chromosome 1 and 2 genome sequences were used to construct a simulated data set of 100 base pair paired-end reads. The simulated read set was constructed with ART version 1.5.0 using the program art_illumina[43]. The simulation parameters used were as follows: data type "paired end", read length "100", fold coverage "100", and quality score "20" (forward and reverse sequence reads). This dataset was used as a benchmark to evaluate the performance of the *de novo* assemblers, gene prediction algorithms, and annotation methods to reproduce the published sequence and annotations of the CMCP6 genome. *V. vulnificus *CMCP6 was recently re-annotated and is regarded as the most complete and accurate of the published *V. vulnificus *genomes at the time of this writing.

### Data cleansing

FastQC was used to evaluate the quality of the sequence reads for each strain[44]. Any repetitive sequence identified by FastQC was removed from the dataset using an in-house perl script. Reads containing 'N' characters were also removed. After the data-cleansing steps were completed we sampled a subset of reads for each strain that was equivalent to 100x coverage based on the Lander and Waterman statistic[30]. After the data-cleansing steps were completed each newly sequenced isolate read set contained 11,400,000 paired reads. In the case of *V. vulnificus *CMCP6, the ART sequencing simulation program art-illumina generated 6,620,286 paired reads for CMCP6 using an identical threshold. This difference may be due to use of an alternative mathematical formula for calculating genome coverage in ART.

### Sequence assembly

Initially, each read set was assembled with VelvetOptimiser version 2.2.0 and Velvet 1.0.17 in order to identify an optimal kmer value for assembly and construct an initial contig set. The optimal kmer values were 79 for *V. vulnificus *CIP8190 and CECT5763, 83 for *V. vulnificus *CMCP6 and 87 for *V. vulnificus *CECT5198, CECT4606, and CECT4886. The VelvetOptimiser parameters were then used to initiate the Velvet assembler. The VelvetOptimiser hash value (kmer) was set to a range of 73 to 93. The read description parameter was set to "-shortPaired". The VelvetOptimiser optimal kmer value was also used as the input kmer value for ABySS version 1.2.6 (abyss-pe) and SOAPdenovo version SOAPdenovo127mer. The default paired-end parameters were used for both assemblers.

### Contig comparison

MuMMER 2.3[31] was used to create sequence alignments between assembled contigs, within collections of assemblies for the same genome and among genomes.

### Genome annotation

*Ab initio *gene-finding and functional annotation for each contig set was performed using the in-house workflow MAP (manuscript in preparation) constructed in the Taverna workflow management system[22]. This workflow executes parallel assembly-to-analysis pipelines on a genomic data set. The *ab initio *annotation methods implemented include Glimmer3.02, GeneMark.hmm and the Rapid Annotation using Subsystem Technology (RAST)[28] web service. The training model used for *ab initio *gene-finding with Glimmer and GeneMark was constructed based on published *Vibrio vulnificus *annotations available in the NCBI database. The RAST web service parameters used were as follows: the genetic code was set to 11 for bacteria, taxonomy id was set to 672 for genus Vibrio, and the corresponding sequencing statistics for each strain were provided to the web service.

### Ortholog identification

OrthoMCL[29]was used to cluster gene predictions with reference genes in the *Vibrio vulnificus *CMCP6 genome. For this application a cluster threshold of 95% identity was used. OrthoMCL[29]was also used to make connections between orthologs among sequenced *Vibrio vulnificus *strains, with a clustering threshold of 70% identity.

### Functional annotation

Gene ontology (GO) terms were assigned using the BLAST2GO software[45]. BLAST2GO was used to perform a BLASTP against the nr (non-redundant) protein database, with e-value cut-off set to 1^E-6^. GO annotations were assigned based on the BLAST2GO database version b2g_mar13. BLAST2GO assigns GO terms based on a weighted system of evidence codes.

### Content and functional comparison

For comparison of assembly-to-annotation workflow outcomes and for comparisons of genomic content, we used the GenoSets software application[33]. The annotations produced by each workflow were loaded into the GenoSets application, which enables comparisons among multiple genomes. Each alternate annotation was treated as a separate "genome" in the GenoSets system. We followed the same gene clustering procedure used in Morrison *et al*. 2012[39] to define sets of genes that differentiate between genomes. To differentiate between the assembly-to-analysis pipeline outcomes, the approach was modified to reflect the expectations that gene sequences arising from different analysis workflows would be highly similar. OrthoMCL clustering was performed against the Vibrio vulnificus reference genome CMCP6 and clusters were formed based on a shared sequence similarity of 90%, instead of the OrthoMCL default parameter of 50%. The increase in stringency to 90% shared sequence similarity results in tightly constrained gene clusters, and allows for the possible of identified genes on the ends of contig that may have not been predicted in their entirety.

## Competing interests

The authors declare that they have no competing interests.

## Authors' contributions

SSM and CJG conceived the study. SSM designed and developed the approaches for the study. SSM and MSJ perform the bioinformatics analysis. RP performed the survey of microbial genome announcements and designed model to keep track of assembly and annotation methods used in survey. SSM, RP, MSJ, CA, FJR, CBA, JDO, and CJG gave final approval of the version to be published. CA and JDO supplied *Vibrio vulnificus *isolates for sequencing. CBA provided *Vibrio vulnificus *sequencing data sets used in this study.

## Supplementary Material

Additional file 1 **Excel document that summarizes the complete list of GO enrichment terms for the workflow description listed in Table 5**. GO enrichment terms were defined as significant with a p-value above .005 cut-off. Click here for file

## Declaration

Funding for this project was provided by NSF SI2:SSE award number 1047896 to CJG and by a DOE GAANN Fellowship for SSM. Programa Consolider-Ingenio 2010 CSD 2009-00006 from MICINN (Spain) provided funding for CA and FJR. The funding agencies had no role in the design, collection, analysis or interpretation of the data, in preparation of the manuscript, or in the decision to submit the manuscript for publication. JDO was supported by the Cooperative State Research, Education, and Extension Service, U.S. Department of Agriculture, Award No. 2009-03571 and any opinion, findings, conclusions, or recommendations expressed in this publication are those of the authors and do not necessarily reflect the views of the U.S. Department of Agriculture.

This article has been published as part of *BMC Genomics *Volume 15 Supplement 8, 2014: Selected articles from the 9th International Symposium on Bioinformatics Research and Applications (ISBRA'13): Genomics. The full contents of the supplement are available online at http://www.biomedcentral.com/bmcgenomics/supplements/15/S8.
